# Colloidal Iron Oxide Formulation for Equine Hoof Disinfection

**DOI:** 10.3390/ani11030766

**Published:** 2021-03-10

**Authors:** Maurizio Isola, Cristina Piccinotti, Massimiliano Magro, Luca Fasolato, Fabio Vianello, Maria Luisa Menandro, Parastoo Memarian, Melissa Rossi, Maria Elena Falomo

**Affiliations:** 1 Department of Animal Medicine, Productions and Health, University of Padova, Viale dell’Università 16, 35020 Legnaro, Italy; cristinapiccinotti.vet@gmail.com (C.P.); marialuisa.menandro@unipd.it (M.L.M.); parastoo.memarian@studenti.unipd.it (P.M.); melissa.rossi@unipd.it (M.R.); mariaelena.falomo@unipd.it (M.E.F.); 2 Department of Comparative Biomedicine and Food Science, University of Padova, Viale dell’Università 16, 35020 Legnaro, Italy; massimiliano.magro@unipd.it (M.M.); luca.fasolato@unipd.it (L.F.); fabio.vianello@unipd.it (F.V.)

**Keywords:** chlorhexidine, povidone-iodine, hoof, horse, pre-surgical disinfection

## Abstract

**Simple Summary:**

Horse hoof possesses a micro-flora that may promote the development of secondary infections following wounds or surgery. For this reason, in vitro and in vivo experiments were used to verify and compare the bactericidal power of two well-known disinfectants, Povidone-iodine (PI) and Chlorhexidine (CHx), and a new stable colloidal suspension of iron oxide, called Iron Animals (IA). In vitro, IA was able to completely suppress the bacterial growth on all of the tested microorganisms. In vivo, PI alone possessed the lowest efficacy for hoof disinfection; CHx resulted as the best treatment in 18% of cases. CHx + IA emerged as the best disinfection protocol for equine hoof achieving the lowest bacterial load in 55% of cases. The addition of IA, after PI or CHx, improved both disinfectants’ effectiveness, leading to the highest bactericidal activity in 82% of cases. IA would deserve a chance as a possible solution in pre-surgical disinfection of the equine hoof, reducing the risk of infection.

**Abstract:**

The presence of bacteria of various origins on horse hoofs enables the onset of infections following trauma or even post-surgical wounds. Thus, the analysis of new antibacterial substances is of fundamental importance. In this study, the antibacterial efficacy of Iron Animals (IA), a stable colloidal suspension of iron oxide, organic acids, and detergents, was tested in vitro and in vivo. In vitro assays were performed to test the unspecific inhibitory effect of IA on both gram-positive and gram-negative bacteria monitoring the microorganism growth by spectrophotometry (optical density OD600) at 37 °C for 24 h. In vivo test consists on the quantification of the bacterial load in colony forming units per gram (CFU/g) of specimens collected from the frog region of the anterior hooves of 11 horses. Sampling followed the application of four disinfectant protocols consisting of two consecutive 3 min scrubs with 50 mL of 10% Povidone-iodine (PI) or 4% Chlorhexidine (CHx), with or without an additional application for 15 min of 10 mL of Iron Animals (PI+IA and CHx+IA). *In vitro*, IA completely suppressed the bacterial growth of all the tested microorganisms, resulting in effectiveness also against CHx-resistant bacteria, such as *Staphylococcus aureus*. In vivo, PI emerged as an ineffective protocol; CHx was effective in 18% of cases, but with the addition of IA (CHx + IA) its use emerged as the best disinfectant protocol for horse hoof, achieving the lowest bacterial load in 55% of cases. The addition of IA, after PI or CHx, improves the effectiveness of both disinfectants leading to the highest bactericidal activity in 82% of cases.

## 1. Introduction

The equine hoof is exposed to a variety of microorganisms, which can be subdivided into (1) skin inhabitants, gram-positive bacteria (*Staphylococcus epidermidis*, *S. aureus*, *α-Streptococcus viridans*, *Micrococcus* spp., *Bacillus* spp., and *Corynebacterium* spp.); (2) gram-negatives of fecal origin (*Escherichia coli*, *Klebsiella pneumoniae,* and *Klebsiella oxytoca)*; (3) hoof-capsule residents (*Acinetobacter* spp.) [1,2,3]. This transient microflora is influenced by the presence of exudate, hair, dirt, and high moisture in the environment [1,4]. 

The disinfection of a wound or a surgical site allows the reduction of the microflora aimed at diminishing the risk of secondary infections. However, hoof disinfection is difficult for the presence of foreign materials, crevices, and the periople, the external lipid-rich layer of the hoof [2,5]. Moreover, the rigidity of the hoof capsule prevents rapid wound closure and healing. For these reasons, diseases involving equine hoof require the removal of foreign materials and, in some cases, the trimming and rasping of the hoof before the application of disinfectants and bandage [2,3,6,7]. 

The most widespread hoof disinfectants remain Povidone-iodine (PI), Chlorhexidine (CHx), iodine tincture, and Lugol solution [3,4,6,8,9,10]. Nevertheless, as antimicrobial resistance represents a growing problem worldwide, alternative strategies are urgently required [11]. In this view, the use of colloidal materials represents a promising option for facing this crucial issue [12]. Among them, considerable experience was developed on iron oxides, with applications in biotechnology and biomedicine [13,14,15,16,17,18]. Based on the authors’ background, a novel unspecific strategy was proposed for eliminating microorganisms and parasites from the animal skin surface, exploiting the redox properties of colloidal iron oxides [19]. The property of iron oxides to produce reactive oxygen species (ROS) was recently reviewed, foreseeing their potential in therapy [20]. 

The final aim of this study is to evaluate the effectiveness of colloidal iron oxides on the enhancement of the bactericidal activity of two common disinfectants (PI and CHx) on the horse hoof.

## 2. Materials and Methods

### 2.1. In Vitro Inhibitory Effect on Bacterial Growth

Standardized inoculum of *Staphylococcus aureus* ATCC 29213, *S. aureus* 393, *Pseudomonas aeruginosa* L2, and *P. aeruginosa* L4L were used for the experiments. The strains were plated on Mueller Hinton Agar (Biolife Italiana srl, Milano, Italy) and incubated at 37 °C for 19 h. Pure colonies were suspended in 10 mL Mueller Hinton Broth (MHB, Biolife Italiana srl, Milano, Italy) and diluted until the concentration was 5.0 Log10 colony forming unit/mL (CFU/mL). The microbroth dilution technique of antimicrobial susceptibility was performed to test the inhibitory effect of the formulations on the bacterial growth in comparison to controls. Dilutions were carried out using sterile deionized water in 96-well flat-bottom microtiter plates and were incubated with standardized inoculum (100 mL). Control experiments on bacteria were carried out by simple addition of sterile deionized water. The microtiter assays were incubated at 37 °C and the optical density (OD_600_), namely the UV-vis absorbance (ABS) at 600 nm, was continuously monitored by spectrophotometry (Multiskan GO Microplate Readers, Thermo Fisher Scientific, Waltham, Massachusetts, USA) for 24 h. Three biological replicates were performed for each strain and two different experiments were duplicated.

### 2.2. Samples Collection

A preliminary experiment was made to set up the study and test the efficacy of Iron Animals (IA) in eliminating microorganisms on the horse hoof. The front and rear hooves of one horse were superficially cleaned to remove the dirt mechanically. A fragment of the left front hoof sole was sampled as control and was subjected to microbiological analysis. The right front hoof sole was treated with two consecutive 3 min scrubs with PI, simulating a pre-surgical scrub. After the scrubs, the sole was rinsed with saline solution and dried with sterile gauze before sampling and analyses. The same experiment was made on the rear hoof replacing the PI scrubs with the application of a gauze soaked in 10 mL of IA on the hoof sole for 15 min. A tissue sample of 2 cm^2^ was collected using a sterile scalpel for microbiological study and then placed in a test tube containing 5 mL of sterile saline solution (0.9% NaCl). In the laboratory, the sample was shaken with vortex and 50 μL of the suspension was used to perform microbiological analysis. The suspension was plated on Nutrient Agar (Oxoid microbiology products, Oxoid Limited, Hampshire, UK) and incubated at 37 °C for 24 h. In the light of the obtained results, which will be discussed subsequently in Section 3.2, the study proceeded as defined in the following protocol.

The study involved 11 horses of different breeds (7–19 years old). The tissues were collected from the frog of the front hooves which, together with the sole, is the most contaminated site of the equine hoof [3]. Horses lived in the same farm and were subjected to the same management; horses were shod every 45–50 days and were housed on rice husk litter cleaned twice a day. Hooves were cleaned once a day with a hoof pick. Only horses with clinically healthy hooves were included in this study.

The left front hoof of each animal was cleaned with a hoof pick and trimmed; then, two consecutive 3 min scrubs were made using a firm sterile brush with 50 mL of 10% PI (Lh Dermoscrub Spugnetta Iodo, Lombardo H, Milan, Italy). At the end of each scrub, the hoof was rinsed with sterile saline solution (0.9% NaCl). The sole was then dried with sterile gauzes and a tissue sample of about 2 cm^2^ was taken from the frog region with the use of a sterile scalpel for microbiological study (protocol PI). After the sampling, 10 mL of Iron Animals (IA, AINT s.r.l. Advanced Iron Nano Technologies, spin-off of the University of Padova, Venice, Italy) were applied and left in place. The sole and the hoof were covered with sterile gauzes, wrapped with Vetrap, and put inside a plastic bag. The top of the bandage was sealed with adhesive tape to avoid any external contamination. Until the application of the bandage, the hoof was held off the ground. After 15 min, the dressing was removed and the sole was rinsed and dried as previously described. The second tissue sample was taken from the frog region (protocol PI+IA). The same protocol was accomplished by replacing the PI with 4% CHx (Lh Dermoscrub Spugnetta Clorexidina 4%, Lombardo H) on the right hooves, setting up protocol CHx (2 scrubs with CHx) and CHx+IA (2 scrubs with CHx + 10 mL of Iron Animals for 15 min).

The collected tissue samples were placed in individual test tubes previously weighed and containing 5 mL of sterile saline solution (0.9% NaCl). In the laboratory, the sample weights were calculated by carrying out a second weighing using a scientific balance (Scientific balance E50S, Gibertini, Milano, Italy) and evaluating the difference in grams between the first weighing of the tube and the second one [2]. Each sample’s weight (range, 30–138 mg) was registered and used to calculate the colony forming units per gram (CFU/g) after the microbiological analyses. Samples were refrigerated at 4 °C and microbiological analyses were performed within 24 h.

### 2.3. Quantitative Microbiology

Each sample, contained in 5 mL of sterile saline solution, was mixed by vortex to spread all the microorganisms in the solution and serial 10-fold dilutions were performed. An aliquot of 100 μL from each dilution was plated on the Nutrient Agar (Oxoid microbiology products, Oxoid Limited, Hampshire, UK) and incubated for 24 h at 37 °C to determine the bacterial counts as the number of CFU/g of the specimen. 

### 2.4. Statistical Analysis

For the preliminary study and in vitro test, descriptive observations were reported. Data obtained by the in vivo sampling and laboratory analyses of quantitative microbiology were performed using the SAS 9.4 software (SAS Institute Inc., Cary, NC, USA). Comparisons among protocols were performed using the non-parametric Kruskal-Wallis test, due to a not-normal distribution of CFU/g. For simplicity, the average CFU/g values obtained from the protocols PI and CHx, in the presence and absence of IA, were normalized for the residual bacterial load achieved in PI protocol, which was used as a reference procedure and was referred to as 100%. The number of cases in which one protocol was better than the others in reducing the residual bacterial load for each horse was analyzed using the Chi-square comparison test between percentages, using the Marascuilo procedure. *p* < 0.05 was considered to be statistically significant.

## 3. Results

### 3.1. Bactericidal Action of IA In Vitro

**Figure 1:**
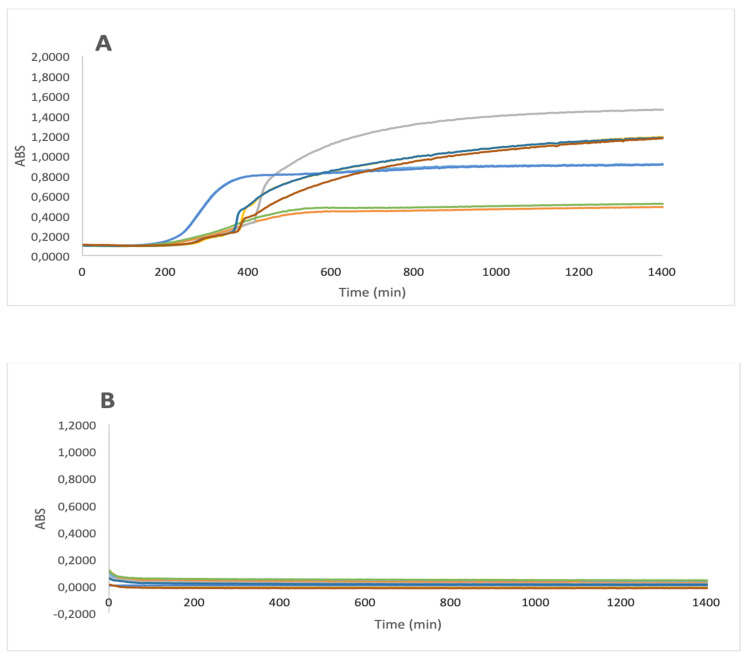
Bacterial growth of *Staphylococcus aureus* ATCC 29,213 (**—**), *S. aureus* 393 (**—**), *Pseudomonas aeruginosa* L2 (**—**), *P. aeruginosa* L4 (**—**) in the absence (**A**) and presence (**B**) of Iron Animals. ABS (arbitrary units) = optical density OD_600_ − blank OD_600_.

The bactericidal effect of IA was tested on both gram-positive and gram-negative bacteria. In Figure 1A, the growth curves of *Staphylococcus aureus* ATCC 29213, *S. aureus* 393, *Pseudomonas aeruginosa* L2, *P. aeruginosa* L4 are presented as controls (broth plus water). It is noteworthy that the application of IA was able to completely suppress the bacterial growth on all the tested microorganisms (Figure 1B). The bactericidal efficacy was definitively substantiated by plate count after bacterial growth, evidencing the complete disappearance of the two microorganisms. The elimination of *S. aureus* is of primary importance in terms of the resistance developed by this microorganism to CHx, which is one of the most widespread disinfectants in clinical practice.

### 3.2. Preliminary Study for the Evaluation of the Bactericidal Action of IA In Vivo

**Figure 2:**
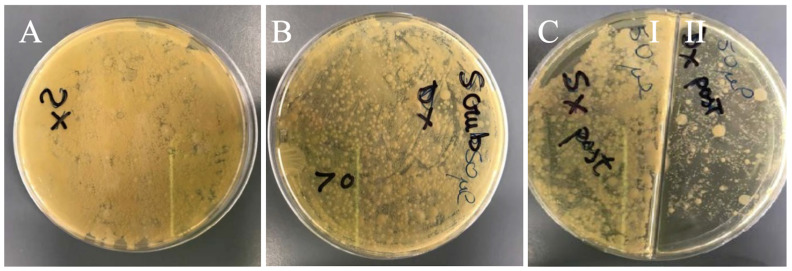
Microbiological analysis of the fragments from the front and rear hoof sole of one horse. The plates show the microbial growth on Nutrient Agar, after incubation at 37 °C for 24 h. (**A**) Sample from the left front hoof sole after superficial cleaning; (**B**) Sample from the right front hoof sole after two consecutive 3 min scrubs with Povidone-iodine (PI); (**C**) I, Sample from the left rear hoof sole after superficial cleaning; II, Sample from the right rear hoof sole after application of 10 mL Iron Animals (IA) for 15 min.

The efficacy of IA in eliminating microorganisms on horse hoof can be appreciated by observing the bacterial growth on Petri dishes. Left front hoof sole was used as control and sampled after a superficial cleaning. Results showed a very high bacterial load from a mixed non-countable microbial flora (Figure 2A). In contrast, the right front hoof sole was treated with two consecutive 3 min scrubs with PI. Moreover, in this second sample, a very high non-countable bacterial load from mixed microbial flora was present (Figure 2B). However, a slightly lower bacterial content, evident from the lighter color, was observed regarding the control fragment (Figure 2A). Figure 2(CI,II) compare the results obtained on the rear hooves. The sample obtained by the left rear hoof sole after a superficial cleaning showed a very high non-countable bacterial load of mixed microbial flora (Figure 2(CI)); while the right rear hoof sole, treated with the application of 10 mL of IA for 15 min (Figure 2(CII)), showed a significantly lower bacterial load and a superior antibacterial effect when compared to the sample treated with PI (Figure 2B).

### 3.3. Comparison of PI and CHx on Microbial Growth of the Horse Hooves in the Presence and Absence of IA

**Figure 3:**
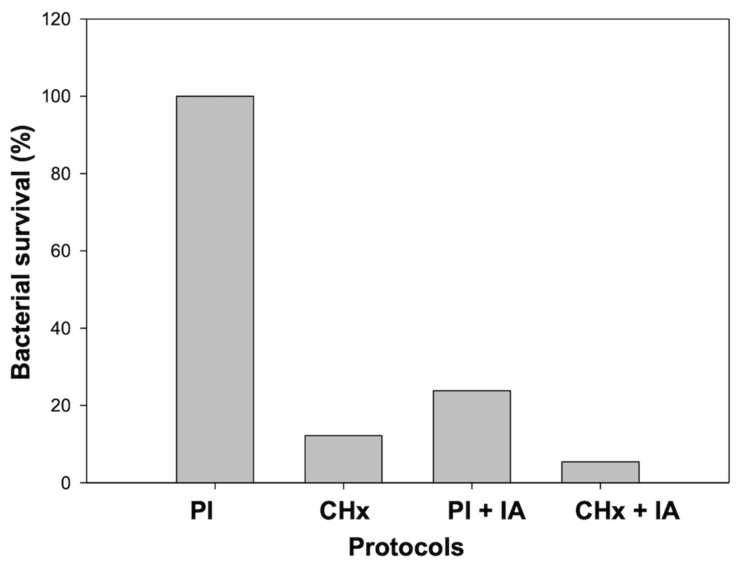
Representation of the average residual bacterial load after different protocols. Data were normalized for the residual bacterial load achieved in Povidone-iodine (PI) protocol, which was used as a reference procedure and referred to as 100%. PI: two consecutive 3 min scrubs with PI; Chlorhexidine (CHx): two consecutive 3 min scrubs with CHx; PI + Iron Animals (IA) and CHx + IA represent PI and CHx protocol followed by the application of 10 mL of IA for 15 min.

As expected, CHx resulted in a significant and more efficient bactericidal activity, leading to seven times lower bacterial load, in comparison to PI (*p* = 0.006) (Figure 3). Interestingly, the application of IA, after PI or CHx, led to a drastic reduction of the bacterial load, although not statistically significant (*p* = 0.12 and *p* = 0.9, respectively). Protocol CHx + IA resulted as the treatment that allows the major reduction of the bacterial load of the equine hoof. Moreover, it resulted in a significant difference from PI treatment (*p* = 0.03).

**Figure 4:**
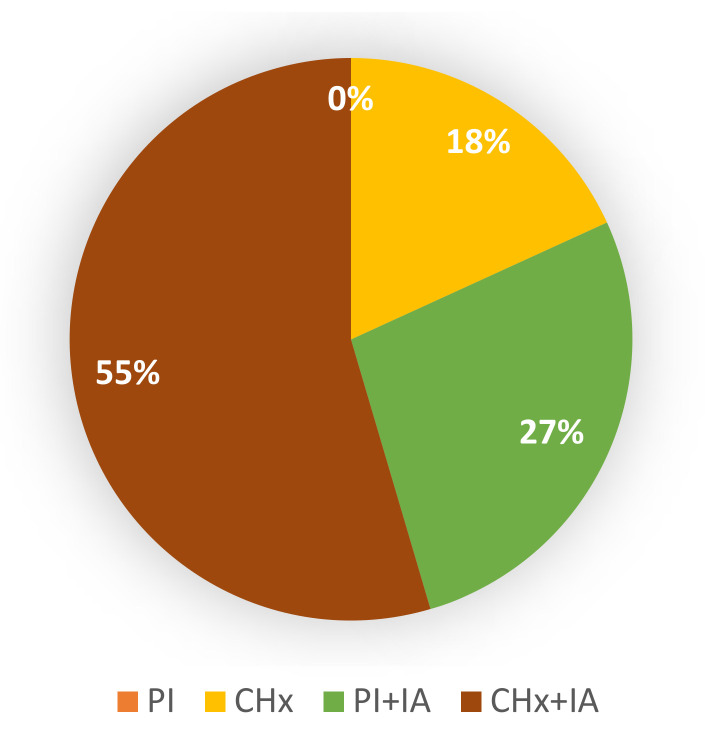
Graphical representation of the fraction of cases in which each protocol obtained the lowest bacterial load within the hooves of the same animal. PI: two consecutive 3 min scrubs with Povidone-iodine (PI); Chlorhexidine (CHx): two consecutive 3 min scrubs with CHx; PI + Iron Animals (IA) and CHx + IA represent PI and CHx protocol followed by the application of 10 mL of IA for 15 min.

A graphical representation of the number of cases, in which each protocol achieved the lowest bacterial load, is presented in Figure 4. The treatment comparison was carried out on the hooves within every single animal. Thus, the variability among different horses was avoided, in terms of both bacterial communities and microbial loads. As shown in Figure 4, a comparison were done between the treatments and their effectiveness in reducing the bacterial load and the results showed that the use of two consecutive 3 min scrubs of PI was not sufficient for correct disinfection of the equine hoof; PI alone was found to be the worst disinfectant treatment (0% of success). The use of CHx alone resulted in the best treatment in 18% of cases. The application of IA, after PI or CHx scrubs, implemented the bactericidal activity of both disinfectants. Indeed, PI + IA and CHx + IA resulted in the best treatments in 27% and 55% of cases, respectively, with a total improvement of 82% of cases following the use of IA.

## 4. Discussion

Given the characteristics of hoof structure and the surrounding environment, the pre-surgical disinfection or cleaning of a wound resulted extremely important. Indeed, the presence of microorganisms on the operative surface or the wound bed is one of the most significant risk factors for the development of secondary infections [4,5]. The most contaminated parts of the equine hoof are the frog and the sole. Moreover, the frog possesses a high content of water and crevices with glands that make the growth of bacteria favorable in a protected environment [3,6]. Nevertheless, to date, no standard protocol for the disinfection of the hoof is provided. Herein, a novel concept of disinfection strategy is reported, employing for the first time a stable colloidal suspension of iron oxide nanoparticles in combination with organic acids and detergents. In order to respect common pre-surgical practices, this patented formulation, commercially termed “Iron Animals (IA)”, was tested in vitro and then examined in vivo after two well-known disinfection protocols (Povidone-iodine: PI, and Chlorhexidine: CHx) on a population of eleven horses. 

In the in vitro study, *Staphylococcus aureus,* gram-positive bacteria was chosen, as it is a matter of concern in the veterinary field and because of its resistance to CHx, which is one of the most common disinfectants in clinical practice. *Pseudomonas aeruginosa* (gram-negative) was chosen to demonstrate the specific mechanism of IA and its efficiency on both gram-positive and gram-negative bacteria. It is noteworthy that, both these species, are commonly associated with tissue infections [21]. The results showed that IA was able to completely suppress the bacterial growth on all the tested microorganisms. Then, we proceeded with a preliminary study that confirmed the possible use of IA on horse hoof and led to exclusion of the use of the rear hooves, as they are more prone to being soiled with fecal material, both because of the conformation of the sole and its anatomical position. Furthermore, the preliminary study highlighted some difficulties in perfectly standardizing the amount of sample collected. These issues have been optimized in order to obtain an in vivo protocol that mimic what would normally occur in pre-surgical scrubs of the hoof or during the cleaning of a hoof wound. For that reason, the study was conducted in the front hooves and it was decided to apply PI and CHx with scrubs carried out through a firm sterile brush. Instead, IA was applied and left in place as the surface was previously clean thanks to the scrubs with PI and CHx. The time of application of IA (15 min) was decided taking into account the length of the entire protocol (scrubs, sampling, application of Iron Animals, bandaging) avoiding procedures that last for a long time and may create unnecessary stress for the animals. In vivo, the use of IA, after PI or CHx, improved the bactericidal activity of both disinfectants.

PI is the most common disinfectant reported in the literature and used in practice for the disinfection of the equine hoof, followed by CHx [9,22]. On the horse hoof, the use of PI for scrubs, but mostly for the creation of cotton soaked with PI solution to be left in place for 24 h, significantly reduced the bacterial load in the different parts of the hoof (wall, sole, and frog). These results were improved by the physical removal of the superficial layer of the hoof [2]. However, Hennig et al. admitted that, despite the use of PI and the removal of the surface tissue of the hoof, the bacterial load of the hoof was still high. Unfortunately, no comparison with CHx was carried out. In contrast with Henning et al., Johnson et al. [3] demonstrated that the use of a 4 min PI scrub decreased the bacterial load of the hoof below the critical upper limit for the reduction of the risk of surgical site infections. Nonetheless, they discouraged the use of PI in direct contact with the skin for several hours due to the appearance of skin lesions and bacterial recolonization of the part [3]. However, it is important to clarify that a minimum threshold of bacterial load for reducing the risk of post-operative infections has never been determined for the hoof. This limit is generally set at 10^5^ microorganisms/g of tissue for surgical skin wounds [3,23], but the composition of the skin and its healing processes are completely different from those of the hoof.

The protocol that involves the addition of Iron Animals to two consecutive 3 min scrubs with Chlorhexidine obtains the highest bactericidal power. This result is statistically significant in comparison to PI scrubs, which represent one of the most used protocols for disinfection during veterinary clinical activity [3]. Moreover, our results are in agreement with other studies comparing PI and CHx, where the latter was significantly more efficient in reducing the bacterial load post scrub [4,8]. Most importantly, the present contribution demonstrates for the first time the possibility of using a metal oxide formulation as a disinfectant in the veterinary field. In the comparison between Chlorhexidine alone and Chlorhexidine plus Iron Animals, the addition of IA does not produce a statistically significant effect; however, the bactericidal activity carries out by IA must not be underestimated. As shown by in vivo test, IA remarkably reduces the bacterial load of the samples. Moreover, as shown by in vitro test, IA is able to eliminate bacteria resistant to Chlorhexidine as the methicillin-resistant *Staphylococcus aureus*. Infections by methicillin-resistant *Staphylococcus aureus* are frequent in the veterinary field and *enterococci* have been isolated from the hands of veterinary surgeons [23]. Indeed, IA was developed for providing a localized unspecific bactericidal action without employing hazardous materials. Specifically, the presented approach is based on the combination of colloidal iron oxides, as local generators of ROS, organic acids (e.g., trichloroacetic acid), and detergents (e.g., sodium dodecyl sulfate). This concept has led to the development of a patented formulation [19], already applied in the field with good results in the treatment of animal skin and hooves. Furthermore, IA showed the ability to quickly clean clogs, frogs, and eliminate skin surface impurities, dead and desquamated cells, and ensuring preventive action for the recurrence of caries at hooves and skin mycoses. It should be mentioned that the product is odorless, does not stain, and is not absorbed by the skin. The latter is a key feature granting the local action of the formulation [19]. The action of the formulation can be explained as the result of the combination of three components: (1) a stable colloidal suspension of iron oxides, which catalytically induce cell death by promoting a localized production of cytotoxic free radicals [24]; (2) a protein precipitating agent, namely 2,2,2-trichloroacetic acid (TCA). Despite the extensive use of TCA as a protein precipitation agent, its molecular mechanism of action remains elusive. It is supposed that TCA leads to protein precipitation by sequestering the water molecules bound to proteins [20]; (3) A denaturing detergent (sodium dodecyl sulfate, SDS), working as membrane disrupting and protein denaturing agent. Its action is due to the disruption of hydrophobic interactions. Furthermore, the detergent allows the dispersion of water-insoluble, hydrophobic compounds into the aqueous medium, including the extraction and solubilization of membrane proteins [25]. In addition, the detergent lowers the surface tension, thus allowing the permeation of the formulation into the hoof porosity, promoting contact with microorganisms, and representing a crucial requirement for the formulation applicability on the animal diseases.

In light of the results obtained so far, further studies are desirable to (i) assess the potential of IA formulation when used alone and against other microorganisms, such as mycosis or yeasts; (ii) optimize the use of Iron Animals both in terms of time and methods of application; (iii) evaluate its use for the antisepsis of surgeon hands that still represent a source of bacterial contamination. Currently, 80% of veterinary surgeons use PI or CHx for pre-surgical hand disinfection and the search for new products, less harmful to the skin, becomes mandatory [26,27].

## 5. Conclusions

Besides potentiating both PI and CHx, the treatment with IA led to the lowest bacterial load in 82% of cases. An application of IA (10 mL, 15 min activity) after two consecutive scrubs with CHx achieved the best bactericidal effect, overcoming the efficiency of the sole CHx. It is important to stress that the resistance of some bacteria, such as *Staphylococcus aureus*, represents a matter of concern that is not solved by the application of common disinfectants such as CHx. In the present study, IA displayed the ability to eliminate *S. aureus*, as substantiated by the in vitro tests. In this light, IA, thanks to its localized and unspecific action, may represent a valid alternative to substances currently used for animal skin disinfection, such as antibiotics. This aspect is of crucial importance considering the implications correlated to the development of microbial resistance. Nevertheless, PI and CHx were used to respect common pre-surgical practices, the observed results stimulate deeper investigations and encourage: (i) the development of protocols based on the sole use of IA, to reveal the actual potential of the proposed approach; (ii) further study on IA in term of time and methods of application.
